# The clinical efficacy and adverse effects of Entecavir plus Thymosin alpha-1 combination therapy versus Entecavir Monotherapy in HBV-related cirrhosis: a systematic review and meta-analysis

**DOI:** 10.1186/s12876-020-01477-8

**Published:** 2020-10-19

**Authors:** Dan Peng, Hai-Yan Xing, Chen Li, Xian-Feng Wang, Min Hou, Bin Li, Jian-Hong Chen

**Affiliations:** Department of Pharmacy, Daping Hospital, Army Medical University, Chongqing, 400042 China

**Keywords:** HBV, Cirrhosis, Entecavir, Thymosin alpha-1, Meta-analysis

## Abstract

**Background:**

Previous studies have demonstrated the benefits of thymosin alpha-1 (Tα1) in anti-virus, immunological enhancement and anti-inflammation. However, it is controversial about the efficacy and safety of entecavir (ETV) plus Tα1 combination therapy versus ETV monotherapy in cirrhotic patients with hepatitis B virus (HBV) infection.

**Methods:**

The systematic review and meta-analysis of randomized clinical trials (RCTs) were performed to evaluate the efficacy and safety of ETV plus Tα1 combination therapy versus ETV monotherapy in HBV-related patients with cirrhosis. We performed a systematic literature search via PubMed, Web of Science, Cochrane Central Register of Controlled Trials (CENTRAL), EMBASE, China National Knowledge Infrastructure (CNKI), Chinese Science and Technology Journals Database (VIP), and Chinese Biological Medicine database (CBM). Relative risk (RR) and standardized mean difference (SMD) with a fixed- or random- effect model were calculated. Heterogeneity was assessed through a Cochrane Q-test and I^2^ values.

**Results:**

Seven RCTs involving 1144 subjects were included in the systematic review and meta-analysis. Compared with ETV monotherapy, ETV plus Tα1 combination therapy led to a higher complete response (RR = 1.18; 95% CI, 1.07–1.30). In post treatment for 24 weeks, the HBV DNA undetectable rate and HBeAg loss rate were higher in ETV plus Tα1 group than in ETV alone group (RR = 1.91; 95% CI, 1.56–2.35; RR = 2.05; 95% CI, 1.62–2.60). However, after 48 and 52 weeks of treatment, there was no significant difference between the combination therapy and ETV monotherapy (RR = 1.07; 95% CI, 0.96–1.18; RR = 1.17; 95% CI, 0.89–1.55). At week 52 of treatment, the HBsAg loss rate of ETV plus Tα1 group was no significance with that of ETV alone group (RR = 1.03; 95% CI, 0.15–7.26). In comparison with ETV alone, the some biochemical parameters and liver fibrosis were obviously improved by ETV plus Tα1, and there was significant heterogeneity. In addition, the number of adverse events was significantly reduced by ETV plus Tα1, compared to ETV alone (RR = 0.48; 95% CI, 0.24–0.95).

**Conclusions:**

ETV plus Tα1 might lead to a higher clinical response and a lower comprehensive adverse reaction rate in HBV-related patients with cirrhosis, compared to ETV alone. However, the whole patients included in this meta-analysis were from Chinese mainland, so that more worldwide RCTs with a larger sample size are needed to verify the current findings.

## Introduction

Liver cirrhosis is an end-stage organic disease characterized as the irreversible fibrosis, necroses of liver cells and multifaceted immune dysfunction [1, 2]. Chronic HBV (CHB) infection is considered as an independent risk factor of the occurrence and progression to cirrhosis. Annually, 2.1–6.0% of HBV-related patients were diagnosed as cirrhosis [3, 4]. Cirrhosis annually caused the death of almost 200,000 patients with CHB, and ranked as the 13th leading cause of death globally [4, 5].

The effective and safe treatment approaches for HBV-related cirrhosis could result in the long-term suppression of HBV, rare and mild adverse effects, restoration of liver function, relief of complications, avoidance of liver transplantation, as well as preventing the occurrence to hepatocellular carcinoma (HCC) and death, in order to improve the quality of life and prolong life expectancy for patients with CHB. The anti-viral treatment to HBV is critical for primary and secondary outcomes of patients with cirrhosis. Meanwhile, HBV-related cirrhosis are commonly accompanied with serious immune dysfunction and chronic systemic inflammation [6]. The present mainstay of anti-HBV treatment is the repression of viral replication with nucleos(t) ide analogs (NAs), such as entecavir (ETV), lamivudine (LAM) and tenofovir disoproxil fumarate (TDF) [7]. In recent years, the immune-mediated agents such as interferon-alpha (IFNα) and Tα1 were also applied for combination therapies of HBV-related patients [8]. Theoretically, the sound immune response is conducive to viral suppression and clearance.

However, NAs alone or its combination treatment for patients with cirrhosis have merely recommended in practice guidelines [2]. IFNα or pegylated interferon-alpha (PegIFNα) is cautiously applied or not recommended as therapeutic drugs for patients with cirrhosis, so as to prevent acute and severe hepatitis. Tα1 or Tα1 plus NAs has been rarely mentioned in these guidelines, which has naturally led to a question that whether Tα1 plus NAs is more favorable than NAs monotherapy.

ETV is one of NAs and approved for listing by FDA in 2005. It is preferentially recommended for treatment to HBV-related patients with cirrhosis owing to its active inhibition of HBV and seldom viral resistance. It was suggested in the previous clinical trials that ETV could be well tolerated by HBV-related patients with compensated or decompensated cirrhosis and was efficacious in improving the virological, biochemical and histological parameters [9–12]. The incidence of drug resistance of ETV was reported only to be 1.2% within 5 years [13].

Tα1 is a synthetic polypeptide consisting of 28 amino acids, which can not only reduce hepatic inflammation, but also promote the maturation in T lymphocytes and the activation of T-helper 1 (Th1) [14, 15]. Tα1 has shown good therapeutic activities and has rarely caused adverse reactions such as transient muscular atrophy, multiple joint pains with edema and rash in patients with viral hepatitis [16–19]. For patients with CHB, Tα1 may lead to a transient increase in their ALT levels, so that it should not be injected to those with symptoms or indications of liver failure. Meanwhile, the clinical trials comparing the efficacy and safety of ETV monotherapy with ETV plus Tα1 combination therapy in HBV-related patients with cirrhosis have been performed in the past 10 years, whereas the results were inconsistent. For example, Jia P and his colleagues found that the serum level of undetectable HBV DNA seemed to be higher in ETV plus Tα1 group than in ETV alone group (sample size 130; RR = 1.82; 95% CI, 1.34–2.48) [20]. However, Xu YQ and his colleagues had the opposite results in undetectable HBV DNA (sample size 60; RR = 0.96; 95% CI, 0.73–1.25) [21].

To figure out the cause of inconsistencies in clinical benefits for HBV-related patients with cirrhosis between ETV monotherapy and ETV plus Tα1 combination therapy, the systematic comparisons between the 2 treatment approaches were needed. Therefore, we conducted a systematic review and a meta-analysis of existing trials to compare the efficacy and safety of ETV alone with ETV plus Tα1 for HBV-related patients with cirrhosis.

## Methods

### Literature search

An electronical search was performed from English-language and Chinese-language databases, including PubMed, Web of Science, Cochrane Central Register of Controlled Trials (CENTRAL), EMBASE, China National Knowledge Infrastructure (CNKI), Chinese Science and Technology Journals Database (VIP) and Chinese Biological Medicine Database (CBM). The following items were applied to search for relevant publications: “Thymosin” OR “Thymosin α1” OR “Thymosin alpha 1”, “Entecavir”, “Hepatitis B” OR “HBV”, “Cirrhosis” OR “Hepatocirrhosis” OR “Posthepatitic cirrhosis”. Furthermore, a manual search of reference lists was conducted to screen out potential eligible clinical trials. The retrieved studies only in abstract form were not systematically evaluated with inadequate data.

### Study selection

The eligible publications comparing the efficacy and adverse effects of ETV plus Tα1 to ETV alone in HBV-related patients with cirrhosis were included in our meta-analyses and systematic review. The inclusion criteria included: (I) HBV-related patients with cirrhosis; (II) randomized controlled trials (RCTs) with a duration of at least 10 weeks and the number of subjects in each group > 10; (III) ETV plus Tα1 as combination therapy group; (IV) ETV monotherapy as the control group. (V) The outcome indexes including at least the following 2 items: Response of subjects including effective response and no response; Virological blood detection such as the rate of undetectable HBV DNA or /and that of HBeAg loss and HBsAg loss; Biochemical and clinical variables reflecting liver function such as the levels of ALT, ALB, A/G, TBIL and AST; and adverse effects including nausea, vomit, allergy and dizziness.

Exclusion criteria were as follows: (I) duplicate literatures; (II) irrelevant topics; (III) reviews; (IV) non-RCT designs; (V) unable to extract the data of HBV-related patients with cirrhosis; (VI) any publications with incomplete data were not available.

### Data extraction

The information was independently extracted by two review authors (D Peng and H. Y Xing) and imported into Review Manager (RevMan, Version 5.3. Copenhagen: The Nordic Cochrane Centre, The Cochrane Collaboration, 2014). Any disagreements about data were resolved through discussing with the corresponding author (J.H Chen). For the studies included, we extracted characteristics of studies, baseline characteristics of subjects, responses of HBV-related patients with cirrhosis, HBV virological responses, changes of biochemical variables and adverse effects of subjects. Characteristics of studies included the first author, regions, study designs, enrollment period, types of diseases, number of patients included, diagnostic criteria of HBC, number of patients in ETV plus Tα1 or ETV alone group, and intervention methods. Baseline characteristics of the subjects were as follows: age, group, gender, ALT, ALB, TBIL, AST and A/G. The HBV virological responses included the undetectable rate of HBV DNA level and the loss rate of HBeAG and HBsAG in serum after treatment. The improvement of hepatic fibrosis was reflected through the serum variables regarding HA, PC-III, LN and C-IV.

### Risk of Bias

Tools of the Cochrane Collaboration were independently applied by two reviewers to summarize the risk of bias among all the studies included. The bias items in each study included were as follows: random sequence generation (selection bias), allocation concealment (selection bias), blinding of participants and personnel (performance bias), blindness of outcome assessment (detection bias), incomplete outcome data (attrition bias), selective reporting (reporting bias), and other bias. Disagreements between two reviewers were settled by consensus or consultation with a third reviewer (C Li).

### Statistical analysis

The meta-analyses were conducted using the Review Manager (version 5.3) software. Dichotomous data were pooled with fixed- or random- effect model and presented as the odds risk (OR) with 95% confidence interval (CI). For the continuous data, mean ± standard deviation (SD) and the number of participants were extracted in each group. Fixed- or random-effect model and standardized mean difference (SMD) with 95% CI were employed for statistics of continuous results. Statistical heterogeneity was assessed through a Cochrane Q-test and *I*^*2*^ values. *P* <  0.1 or *I*^*2*^ > 50% were represented for significant heterogeneity among the trials included. Sensitivity, subgroup and meta-regression analyses were performed to investigate the source of heterogeneity in each study. Subgroup analyses were performed according to the duration of treatment. In meta-regression analyses, the covariates included the years of publication, types of cirrhosis and diagnostic criteria for cirrhosis. The publication bias was evaluated with funnel plots. Besides from Cochran’s Q-test, *P* <  0.05 was expressed as a significant difference among the analyzed studies.

## Results

### Characteristics of studies

**Fig. 1:**
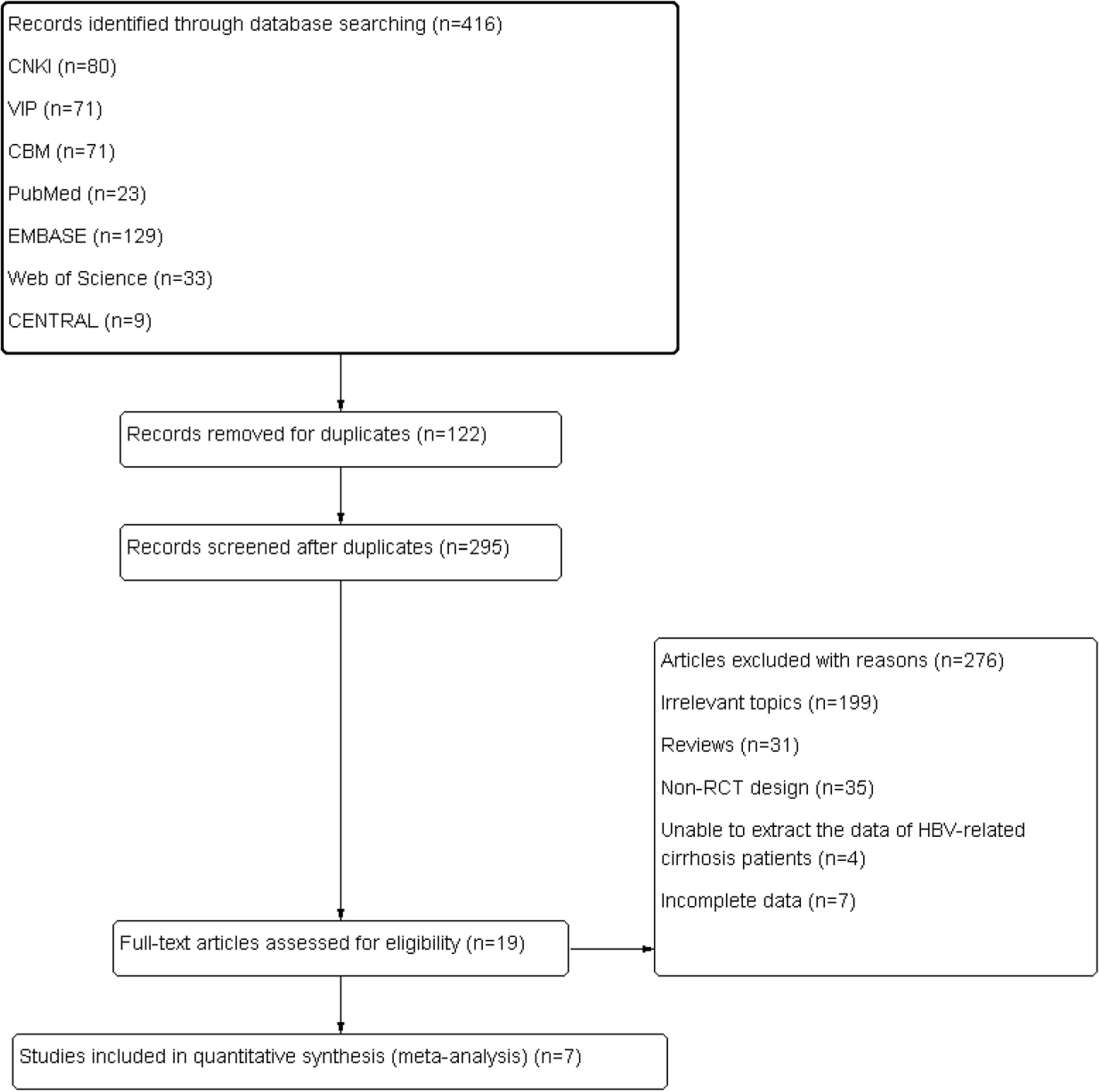
Flow chart for selection of relevant publications. The figures represent the number of articles included per stage

We totally identified 416 publications through database searching. Seven studies were lastly included for our meta-analyses [20–26] (Fig. 1). The types of disease included HBV-related cirrhosis (HBC), compensated HBV-related cirrhosis (CHBC) and decompensated HBV-related cirrhosis (DHBC). Only two studies contained patients with the unclassified types of HBC. Patients with DHBC were pointed out in one study, and patients with CHBC were mentioned in other studies included. Three studies were based on Guidelines for Prevention and Treatment of Chronic Hepatitis B in 2010 or 2015, and only one study followed the Diagnostic Criteria of HBV in 2000. Diagnostic criteria were not mentioned in 3 other studies. ETV (0.5 mg) per day were orally administrated by the subjects of monotherapy group. In combination therapy group, ETV (0.5 mg) per day were orally administrated and Ta1 (1.6 mg) subcutaneously injected twice per week. The detailed characteristics of studies were summarized in Table 1.

**Table 1:** Characteristics of the included studies

Author [year]	Region	Study design	Enrollment period	Type of disease	Number of patients included	Diagnostic criteria of HBV	Groups	Number of patients in control/ETV plus Tα1 (n)	Intervention method
Shi TM [2013] [25]	Gansu Province, Jiayuguan	RCT	2009–2012	HBC	30	Diagnostic Criteria of HBV revised by Xian National Viral Hepatitis Conference in 2000	CG VS. EG	15 15	ETV 0.5 mg, once per day, treated for 48 weeks. ETV 0.5 mg, once per day, combined with Tα1 injection 1.6 mg, twice per week, treated for 24 weeks, and then single ETV 0.5 mg, once per day, treated until 48 weeks.
Diao YH [2017] [22]	Henan Province, Nanyang	RCT	2013.4-2014.4	CHBC	80	Guidelines for prevention and treatment of chronic hepatitis B in 2010	CG VS. EG	40 40	ETV 0.5 mg, once per day, treated for 24 weeks. ETV 0.5 mg, once per day, combined with Tα1 injection 1.6 mg, twice per week, treated for 24 weeks.
Wu XN [2018] [23]	China	RCT	2013.01-2015.09	CHBC	690	NA	CG VS. EG	339 351	After 26 weeks of ETV treatment (0.5 mg per day), patients were randomly assigned to receive ETV (0.5 mg per day) or combination with Tα1 injection (1.6 mg twice per week) for 52 weeks.
Wang XR [2016] [26]	Hei longjiang Province, Jiamusi	RCT	2013.4- 2016.4	HBC	50	NA	CG VS. EG	25 25	ETV 0.5 mg, once per day, treated for 12 weeks. ETV 0.5 mg, once per day, combined with Tα1 injection 1.6 mg, twice per week, treated for 12 weeks.
Xu YQ [2017] [21]	Sichuan Province, Leshan	RCT	2014.1- 2016.12	DHBC	60	NA	CG VS. EG	30 30	ETV 0.5 mg, once per day, treated for 48 weeks. ETV 0.5 mg, once per day, combined with Tα1 injection 1.6 mg, twice per week, treated for 48 weeks.
Zhang XX [2018] [24]	Liaoling Province, Shenyang	RCT	2014..5- 2017.5	CHBC	104	Guidelines for prevention and treatment of chronic hepatitis B in 2010	CG VS. EG	52 52	ETV 0.5 mg, once per day, treated for 24 weeks. ETV 0.5 mg, once per day, combined with Tα1 injection 1.6 mg, twice per week, treated for 24 weeks.
Jia P [2017] [20]	Heilong jiang Province, Jiamusi	RCT	2016.5- 2017.5	CHBC	130	Guidelines for prevention and treatment of chronic hepatitis B in 2015	CG VS. EG	65 65	ETV 0.5 mg, once per day, treated for 6 months. ETV 0.5 mg, once per day, combined with Tα1 injection 1.6 mg, twice per week, treated for 6 months.

*CG* control group, the group with ETV monotherapy, *EG* experimental group, the group with ETV plus Tα1 combination therapy, *RCT* randomized controlled trials, *HBC* HBV-related cirrhosis, *CHBC* compensated HBV-related cirrhosis; DHBC, decompensated HBV-related cirrhosis; NA, not available

### Characteristics of patients

Five of 7 studies included the mean age of subjects, which ranged from 32 to 69 years old. Gender was provided in 6 studies, of which the percentage of males ranged from 43.3 to 76.0% in the ETV alone group, and it was from 50.0 to 80.0% in ETV plus Tα1 group. At least three baseline measures of ALT, TBIL, AST, ALB and A/G were mentioned in each study. Characteristics of patients were summarized in Additional file 5: Table S1.

### Risk of bias

In the assessment of random sequence generation, 3 studies had a low risk. The allocation concealment, performance bias and detection bias remained unclear. All bias items of incomplete outcome data and 85% (6/7) bias items of selective reporting had a low risk. Other risks of bias were not estimated becsuse of their inefficient information in each study included. The judgements about each risk of bias items presented as percentages across all studies included were summarized in Additional file 1: Fig. S1.

### Complete and no response

**Fig. 2:**
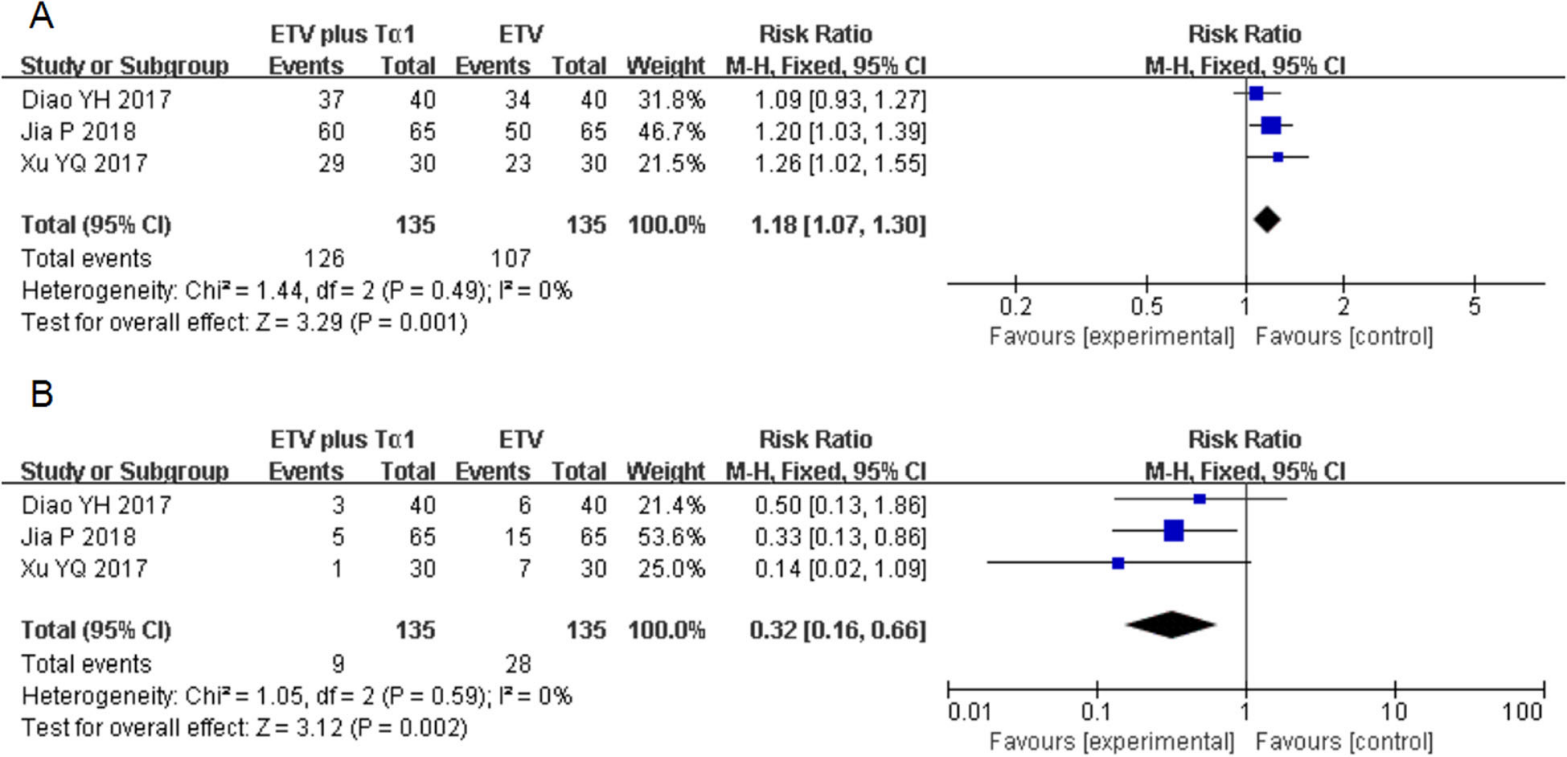
Relative risk of the efficacy to HBV-related patients with cirrhosis in ETV plus Tα1 group and ETV alone group. **a** meta-analysis for the effective response; **b** meta-analysis for no response. Blue boxes indicate the dichotomous data in the forest plots. CI, confidence interval; M-H, Mantel-Haensel

Three studies with 270 subjects were included in the meta-analysis with regarding complete and no response. A higher complete response was observed in the combination therapy group (RR = 1.18; 95% CI, 1.07–1.30, *P* = 0.001). No significant heterogeneity was found between the two treatment groups (*P* = 0.49, *I*^*2*^ = 0%) (Fig. 2a). Furthermore, the rate of no response in the ETV plus Tα1 group was significantly lower than that of ETV alone group (RR = 0.32; 95% CI, 0.16–0.66, *P* = 0.002). No significant heterogeneity was observed in the two therapies (*P* = 0.59, *I*^*2*^ = 0%) (Fig. 2b).

### The HBV DNA undetectable rate

**Fig. 3:**
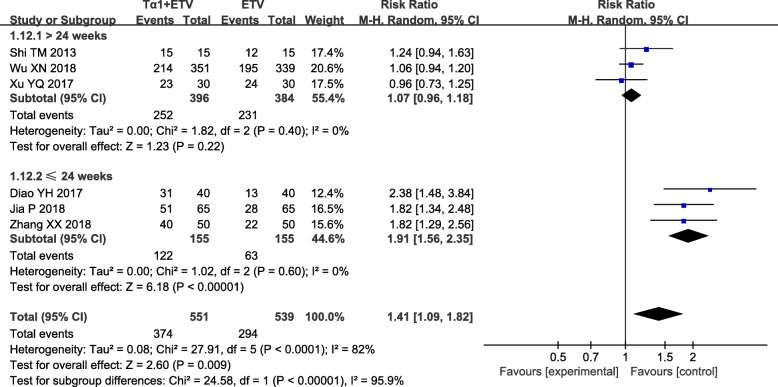
Summary of pooled results about the HBV DNA undetectable rate in ETV plus Tα1 group and ETV alone group. The duration of treatment in subgroup analyses included both less than or equal to 24 weeks and more than 24 weeks. “Events” represents the number of subjects undetected with HBV DNA. “Total” represents the number of subjects in that group. “Test for overall effect” represents the pooled estimate of risk ratio after comprehensive analysis of all studies. Blue boxes indicate the dichotomous data in the forest plots. CI, confidence interval; M-H, Mantel-Haensel

In the meta-analysis, six studies reported the virological response of 1090 patients after treatment of 24 weeks, 48 weeks and 52 weeks. We divided the eligible six studies into two subgroups. Subgroup 1 contained four trials with 310 patients (155 in ETV plus Tα1 group and 155 in ETV alone group) treated for 24 weeks. The sub-analysis suggested that the HBV DNA undetectable rate of the combination therapy was higher than that of ETV monotherapy (RR = 1.91; 95% CI, 1.56–2.35, *P* <  0.00001) (Fig. 3a and b). There was no significant heterogeneity in subgroup 1 (*P* = 0.40, *I*^*2*^ = 0%).

Subgroup 2 contained three studies with 780 patients (396 in ETV plus Tα1 group and 384 in ETV alone group) treated for 48 and 52 weeks. The sub-analysis indicated that the HBV DNA undetectable rate in ETV plus Tα1 group was no significant difference with ETV alone group (RR = 1.07; 95% CI, 0.96–1.18, *P* = 0.22) (Fig. 3 and Additional file 2: Fig. S2). There was no significant heterogeneity in subgroup 2 (*P* = 0.35, *I*^*2*^ = 0%).

### The HBeAg loss rate

**Fig. 4:**
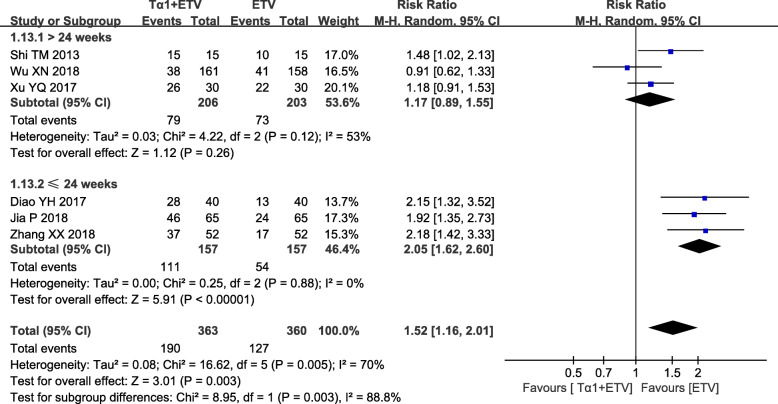
Summary of pooled results about the HBeAG loss rate in ETV plus Tα1 group and ETV alone group. The duration of treatment in subgroup analyses included both less than or equal to 24 weeks and more than 24 weeks. “Events” represents the number of subjects experiencing a HBeAG loss. “Total” represents the number of subjects in that group. “Test for overall effect” refers to the pooled estimate of risk ratio after comprehensive analysis of all studies. Blue boxes indicate the dichotomous data in the forest plots. CI, confidence interval; M-H, Mantel-Haensel

Seven hundred twenty-three subjects were involved in the six studies, through which the HBeAg loss rate was reported. The heterogeneity of overall tests was significant so that random-effect model was used to analyze the overall effects (*P* = 0.005, *I*^*2*^ = 70%). The HBeAg loss rate of the combination therapy group was higher than that of the monotherapy group among those studies (RR = 1.52; 95% CI, 1.16–2.01, *P* = 0.003) (Fig. 4 and Additional file 3: Fig. S3).

In the subgroup analyses, the duration of treatment included 24 weeks, 48 weeks and 52 weeks. The results of Subgroup 1, which contained three studies with 314 patients treated for 24 weeks, reported that the HBeAg loss rate of ETV plus Tα1 group was greater than that of ETV alone group (RR = 2.05; 95% CI, 1.62–2.60, *P* <  0.00001). There was a significant heterogeneity (*P* = 0.12, *I*^*2*^ = 53%). Another three studies involving 409 patients treated for 48 and 52 weeks were included in the Subgroup 2. The results showed that the HBeAg loss rate was similar between the two groups (RR = 1.17; 95% CI, 0.89–1.55, *P* = 0.26). There was no significant heterogeneity (*P* = 0.88, *I*^*2*^ = 0%). (Fig. 4 and Additional file 3: Fig. S3).

### The HBsAg loss rate

**Fig. 5:**
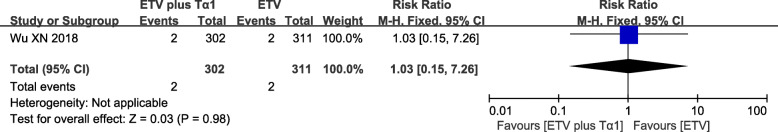
Summary of pooled results including the HBsAG loss rate in ETV plus Tα1 group and ETV alone group. “Events” represents the number of subjects experiencing a HBsAG loss. “Total” represents the number of subjects in that group. “Test for overall effect” represents the pooled estimate of risk ratio after comprehensive analysis of all studies. Blue boxes indicate the dichotomous data in the forest plots. CI, confidence interval; M-H, Mantel-Haensel

Only one trial including 615 subjects reported the HBsAg loss rate in post-treatment [23]. After the treatment for 52 weeks, the HBsAg loss rate of ETV plus Tα1 group was no significance with that of ETV alone group (RR = 1.03; 95% CI, 0.15–7.26, *P* = 0.98) (Fig. 5).

### Biomedical and clinical variables

Biomedical and clinical variables, including ALB, AST, ALT, TBIL and A/G, were extracted from six eligible studies involving 454 participants. The results of meta-analyses were summarized in Table 2. Serum levels of ALB, AST, ALT, TBIL and A/G were significantly enhanced after treatment with ETV plus Tα1 or ETV alone. Compared with ETV alone, ETV plus Tα1 significantly increased the levels of AST and ALT, while there was no obvious difference in the serum levels of ALB, TBIL and A/G. The significant heterogeneity was found in the most meta-analyses regarding biomedical and clinical variables. The results of sensitivity analyses were summarized in Table S2*.* Heterogeneity remained significant.

**Table 2:** Summary of pooled results including biochemical variables

Variable	Studies included (n)	Patients included (n)	SMD	95%CI	Significance, *P*	Heterogeneity	
						*P*	*I*^*2*^
ALB							
After treatment, EG vs. CG	2	90	−0.38	−2.12, 1.37	0.67	0.0001	93%
EG, before vs. after	2	90	−1.62	− 2.87, − 0.36	0.01	0.02	81%
CG, before vs. after	2	90	−1.37	−2.05, −0.68	< 0.0001	0.15	51%
AST							
After treatment, EG vs. CG	4	364	−1.33	− 1.59, − 1.06	< 0.00001	0.31	14%
EG, before vs. after	4	364	8.89	3.45, 14.32	0.001	< 0.00001	99%
CG, before vs. after	4	364	7.56	2.47, 12.64	0.004	< 0.00001	99%
ALT							
After treatment, EG vs. CG	6	454	−1.12	−1.70, −0.55	0.0001	< 0.00001	87%
EG, before vs. after	6	454	10.45	5.83, 15.08	< 0.00001	< 0.00001	99%
CG, before vs. after	6	454	11.61	6.69, 16.53	< 0.00001	< 0.00001	99%
TBIL							
After treatment, EG vs. CG	6	454	−0..35	1.22, 0.53	0.44	< 0.00001	95%
EG, before vs. after	6	454	3.31	1.28, 5.34	0.001	< 0.00001	98%
CG, before vs. after	6	454	3.26	1.54, 4.99	0.0002	< 0.00001	97%
A/G							
After treatment, EG vs. CG	3	314	0.47	−0.88, 1.82	0.49	< 0.00001	97%
EG, before vs. after	3	314	−1.26	−2.19, −0.33	0.008	< 0.00001	93%
CG, before vs. after	3	314	−1.06	−1.30, −0.83	< 0.00001	0.61	0%

*CG* control group, the group with ETV monotherapy, *EG* experimental group, the group with ETV plus Tα1 combination therapy, *SMD* standardized mean difference, *CI* confidence interval, *ALT* alanine aminotransferase, *ALB* albumin, *TBIL* total bilirubin; AST, aspartate aminotransferase; A/G, the albumin globulin ratio

The results of meta-regression analyses were summarized in Table S3. Heterogeneity about serum ALT was relevant with the criteria of diagnostic in ETV plus Tα1 combination group or ETV alone group (*P* = 0.150; *P* = 0.050). Heterogeneity about serum TBIL was not related to the criteria of diagnostic, year of publication or types of cirrhosis in ETV plus Tα1 combination group or ETV alone group.

### Serum variables about hepatic fibrosis

Liver fibrosis is a prominent pathological feature in patients with cirrhosis. Serum levels of HA, PC-III, LN and C-IV have an impact on the synthesis and degradation about collagen, proteoglycan and glycoprotein in liver extracellular matrix. In this meta-analysis, serum variables about hepatic fibrosis were only reported one trial involving 120 subjects, including HA, PC-III, LN and C-IV. Besides of the serum LN level, we found that serum levels of HA, PC-III and C-IV in patients treated with ETV plus Tα1 were obviously decreased in comparison with ETV alone (Table 3). The more RCTs about the effect of ETV plus Tα1 combination therapy on liver fibrosis in HBV-related cirrhosis should be conducted to test the results.

**Table 3:** Summary of pooled results regarding serum variables about hepatic fibrosis

Variable	Studies included (n)	Patients included (n)	SMD	95%CI	Significance, *P*	Heterogeneity	
						*P*	*I*^*2*^
HA							
After treatment, EG vs. CG	1	114	−2.38	−2.87, −1.89	< 0.00001	NA	NA
PC-III							
After treatment, EG vs. CG	1	114	−2.92	−4.42, −1.43	< 0.00001	NA	NA
LN							
After treatment, EG vs. CG	1	114	−1.99	−4.50, 0.52	0.06	NA	NA
C-IV							
vAfter treatment, EG vs. CG	1	114	−2.60	−3.86, −1.33	< 0.0001	NA	NA

*CG* control group, the group with ETV monotherapy, *EG* experimental group, the group with ETV plus Tα1 combination therapy, *SMD* standardized mean difference, *CI* confidence interval, *HA* hyaluronic acid, *PC-III* precollagen III, *LN* laminin, *C-IV* type IV collagen, *NA* not applicable

### Adverse events

**Fig. 6:**
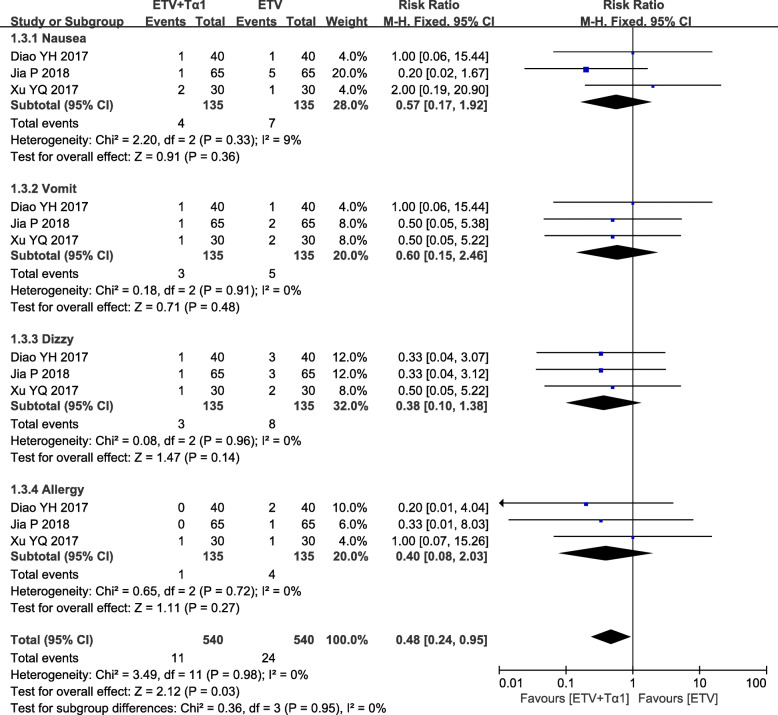
Summary of pooled results including adverse advents in ETV plus Tα1 group and ETV alone group. Meta-analysis for the incidence of adverse reactions included nausea, vomit, allergy or dizziness. “Test for overall effect” represents pooled estimate of risk ratio after comprehensive analysis of all studies. Blue boxes indicate the dichotomous data in the forest plots. CI, confidence interval; M-H, Mantel-Haensel

It was reported in three eligible studies involving 270 subjects that 35 patients experienced the adverse events including nausea, vomit, allergy and dizziness. The results of meta-analysis showed that ETV plus Tα1 combination therapy led to a significant decrease in the number of adverse events, compared with ETV monotherapy (RR = 0.48; 95% CI, 0.24–0.95, *P* = 0.03) (Fig. 6 and Additional file 4: Fig. S4). No significant heterogeneity was found (*P* = 0.98, *I*^*2*^ = 0%). However, no significant difference was observed between the two groups in nausea, vomit, allergy or dizziness, respectively.

## Discussion

Patients with CHB commonly experienced liver fibrosis, cirrhosis, HCC and death as the progression of disease [27, 28]. Although the current expert consensus showed that the effective anti-viral treatment to HBV is vital for improving prognoses and preventing complications in patients with cirrhosis, treatments for adverse reactions, immunosuppression and chronic systemic inflammation should not be ignored [29, 30]. In this meta-analysis, we assessed the efficacy and safety of ETV plus Tα1 combination therapy versus ETV monotherapy for HBV-related patients with cirrhosis. Seven studies conformed to the inclusion criteria. Our results showed that ETV plus Tα1 could obviously increase the complete response and contribute to the reduction of adverse reactions, in comparison with ETV alone. Subgroup analysis about types of adverse reactions indicated that ETV combined with Tα1 had no significance with ETV alone in nause, vomit, dizziness or allergy, respectively.

Rehermann B and his colleagues supported that the inhibition and elimination of HBV depended mainly on the potent and diverse immune responses of T cells in host [31]. It was showed in vitro experiments that Tα1 could inhibit the production of inflammatory cytokines such as TNF-α and potentiate immunocellular function via inducing the maturation of T-cells and up-regulation of CD4+, CD8+ T cells and natural killer (NK) cells [32]. It was verified in vivo experiments that Tα1 was associated with the activation of NK cells in patients with CHB [8]. Our results demonstrated that ETV plus Tα1 combination therapy could provide additional benefits in the inhibition of HBV DNA and negative conversion of HBeAg over ETV monotherapy, but the negative conversion rate of HBsAg was similar between the 2 treatment approaches. Substantial heterogeneity was observed in undetectable HBV DNA and HBeAg negative conversion rate (*I*^*2*^ > 50%), and subgroup analyses showed that the duration of treatment could be an important factor of primary results for HBV-related patients with cirrhosis.

The current meta-analysis showed that the combination therapy during Tα1 add-on significantly improved the serum biochemical variables including ALT, ALB, AST, TBIL and A/G were, compared with ETV monotherapy. The results indicated that the combination treatment had a better effect on the improvement in the function of hepatocytes and remission of hepatic damage. Yang XL [15] found that Tα1 could protect liver of rat against damages via down-regulating TNF-α and up-regulating IL-10, and result in a relief of hepatic inflammation and hepatocyte apoptosis, which was also in line with our findings.

HBV-related cirrhosis is an important stage of progressive liver injuries or fibrosis [33]. Recovery of cirrhosis was related with the degradation of fibrous septa, regeneration of hepatocytes to replace fibrotic tissues and restoration of a lobular architecture [34, 35]. We found that serum variables of hepatic fibrosis including HA, PC-III, LN and C-IV were significantly reduced in ETV plus Tα1 group, compared with ETV alone group. The results reminded that the combination treatment during Tα1 add-on induced the more degradation of fibrous scar and less deposition of excessive extracellular matrix (ECM), compared with ETV alone. However, the trials included was short course of treatment and small sample size, which could not completely present the potential differences in clinical benefits between the 2 treatment approaches. More large, long-term and high-quality studies were still being executed.

In addition, HCC and the complications of cirrhosis include variceal hemorrhage, portal hypertension, ascites, spontaneous bacterial peritonitis and hepatic encephalopathy, which have an important impact on the expected life of patients [27, 36]. Although these clinical outcomes were not systematically reviewed, one study included in this meta-analysis reported that ETV plus Tα1 combination group had a lower incidence of HCC after treatment of 51 weeks, and yet had no benefits in clinical outcomes regarding ascites, hepatic encephalopathy, variceal hemorrhage and liver stiffness, in comparison with ETV alone group [26]. The results were consistent with the study of Liang YR, which indicated that Tα1 therapy improves liver function and obviously extend recurrence-free and overall survival for patients with HBV-related HCC [37]. Unfortunately, the participants of RCTs were from Chinese mainland. With a view to the diversities among people of different races and regions, more global multicenter randomized double-blind trials will need to be performed.

There were other possible limitations in this meta-analysis. Firstly, the diagnostic criteria of HBV-related cirrhosis were inconsistent among the studies included, and the severity of patients with cirrhosis were not exactly the same. Secondly, the characteristics of subjects included were incomplete in some studies, and the statistical sample size of some trials was too small to adequately compare the differences in clinical benefits between monotherapy and combination therapy. Thirdly, heterogeneity was remarkable in biochemical and virological variables. Despite subgroup, sensitivity and meta-regression analyses were detected in this meta-analysis, the resources of heterogeneity were not well clarified. Fourthly, individuals included in RCTs were all from China, international multicenter studies were needed to verify our results.

## Conclusions

This meta-analysis indicated that ETV plus Tα1 combination therapy was safer and more effective than ETV monotherapy for HBV-related patients with cirrhosis. However, our observations were better for Chinese, which were unable to be generalizable in global countries, so that international, large and well-designed multicenter RCTs needed to be performed.

## Supplementary information

**Additional file 1: Fig. S1.** Risk of bias assessment.

**Additional file 2: Fig. S2.** The funnel plot of treatment duration in evaluation for the HBV DNA undetectable rate in ETV plus Tα1 group and ETV alone group.

**Additional file 3: Fig. S3.** The funnel plot of treatment duration in evaluation for the HBeAG loss rate in ETV plus Tα1 group and ETV alone group.

**Additional file 4: Fig. S4.** The funnel plot of effect estimate against its standard error to adverse advents.

**Additional file 5: Table S1.** Characteristic of the patients included.

**Additional file 6: Table S2.** Sensitivity analyses including biochemical variables.

**Additional file 7: Table S3.** Summary of pooled results regarding meta-regression.

## Data Availability

All data and materials were presented within the manuscript and additional supporting files.

## Abbreviations

HBV: Hepatitis B virus
RCTs: Randomized clinical trials
RR: Relative Risk
SMD: Standardized mean difference
CHB: Chronic hepatitis B virus
HCC: Hepatocellular carcinoma
NAs: Nucleos(t) ide analogs
CI: Confidence interval
ALT: Alanine Aminotransferase
ALB: Albumin
TBIL: Total bilirubin
AST: Aspartate aminotransferase
A/G: the albumin globulin ratio
HA: Hyaluronic acid
PC-III: Precollagen III
LN: Laminin
C-IV: Type IV Collagen
