# Supplementary material for: The clinical efficacy and adverse effects of Entecavir plus Thymosin alpha-1 combination therapy versus Entecavir Monotherapy in HBV-related cirrhosis: a systematic review and meta-analysis
Source: BMC Gastroenterol. 2020 Oct 19;20:348. doi: 10.1186/s12876-020-01477-8 (PMC7574490; doi:10.1186/s12876-020-01477-8)
Supplement: Supplementary file 5 — Additional file 5: Table S1. Characteristic of the patients included. [file 12876_2020_1477_MOESM5_ESM.doc]

**Table S1. Characteristic of the patients included**

| **Author**  **[year]** | **Groups** | **Ages** | **Gender**  **(man/female)** | **ALT**  **(U/L)** | **ALB**  **(g/L)** | **TBIL (μmmol/L)** | **AST**  **(U/L)** | **A/G** |
| --- | --- | --- | --- | --- | --- | --- | --- | --- |
| Shuai TM  [2013] | CG  EG | NA  NA | 11/4  10/5 | 162.3±18.2  168±15.1 | 32.1±1.6  31.2±1.5 | 72.6±16.4  73.3±26.4 | NA  NA | NA  NA |
| Wang XR  [2018] | CG  EG | 52.88±10.15  53.64±8.42 | 19/6  20/5 | 105.80±77.04  115.16±86.01 | NA  NA | 24.58±15.45  28.19±13.30 | 87.40±64.26  98.68±47.33 | NA  NA |
| Diao YH  [2017] | CG  EG | NA  NA | NA  NA | 286.28±13.20  288.70±18.46 | NA  NA | 29.64±3.06  29.30±2.77 | 93.35±8.10  93.46±9.30 | 1.43±0.23  1.44±0.18 |
| Xu YQ  [2017] | CG  EG | 47.3±5.2  46.6±5.2 | 13/17  15/15 | 95±34  96±33 | 31±8  32±9 | 31.2±12.8  32.6±11.9 | NA  NA | NA  NA |
| Zhang XX  [2018] | CG  EG | 48.0±6.1  48.3±5.9 | 33/19  33/19 | 268.51±15.67  266.84±16.25 | NA  NA | 29.45±3.38  29.96±3.57 | 92.11±2.05  92.62±2.34 | NA  NA |
| Jia P  [2018] | CG  EG | 40.8±6.6  42.5±6.2 | 39/26  38/27 | 287.65±13.27  285.79±12.67 | NA  NA | 29.59±3.23  29.66±3.12 | 93.57±7.48  93.24±7.63 | 1.44±0.20  1.45±0.21 |
| Wu XN  [2018] | CG  EG | 46.3±9.7  46.1±10.7 | 254/83  276/75 | 52.0 (34.2, 79.5)  54.0 (36.0, 93.2) | 41.2 (37.0, 44.4)  42.0 (37.3, 45.1) | NA  NA | 46.0 (33.3, 76.3)  46.0 (35.0, 76.5) | NA  NA |
| CG (control group), the group with ETV monotherapy; EG (experimental group), the group with ETV plus Tα1 combination therapy; NA, not available; ALT, alanine aminotransferase; ALB, albumin; TBIL,total bilirubin; AST, aspartate aminotransferase; A/G, the albumin globulin ratio. | | | | | | | | |
