# Supplementary material for: The clinical efficacy and adverse effects of Entecavir plus Thymosin alpha-1 combination therapy versus Entecavir Monotherapy in HBV-related cirrhosis: a systematic review and meta-analysis
Source: BMC Gastroenterol. 2020 Oct 19;20:348. doi: 10.1186/s12876-020-01477-8 (PMC7574490; doi:10.1186/s12876-020-01477-8)
Supplement: Supplementary file 6 — Additional file 6: Table S2. Sensitivity analyses including biochemical variables. [file 12876_2020_1477_MOESM6_ESM.doc]

| **Table S2 Sensitivity analyses including biochemical variables** | | | | | | | |
| --- | --- | --- | --- | --- | --- | --- | --- |
| Variable | Studies included (n) | Patients included (n) | SMD | 95%CI | Significance, *P* | Heterogeneity | |
|  |  |  |  |  |  | *P* | *I^2^* |
| AST | | | | | | | |
| After treatment, EG vs. CG | 3 | 284 | -1.37 | -1.80, -0.94 | <0.00001 | 0.14 | 55% |
| EG, before vs. after | 3 | 284 | 9.25 | 1.82, 16.67 | 0.01 | <0.00001 | 99% |
| CG, before vs. after | 3 | 284 | 7.51 | 0.87, 14.15 | 0.03 | <0.00001 | 99% |
| ALT | | | | | | | |
| After treatment, EG vs. CG | 5 | 374 | -1.00 | -1.67, -0.33 | 0.004 | <0.00001 | 88% |
| EG, before vs. after | 5 | 374 | 9.25 | 4.63, 13.87 | <0.0001 | <0.00001 | 99% |
| CG, before vs. after | 5 | 374 | 9.74 | 4.93, 14.55 | <0.0001 | <0.00001 | 99% |
| TBIL | | | | | | | |
| After treatment, EG vs. CG | 5 | 374 | -0.59 | -1.44, 0.25 | 0.17 | <0.00001 | 93% |
| EG, before vs. after | 5 | 374 | 2.95 | 0.74, 5.16 | 0.009 | <0.00001 | 98% |
| CG, before vs. after | 5 | 374 | 2.80 | 1.03, 4.58 | 0.002 | <0.00001 | 97% |
| A/G | | | | | | | |
| After treatment, EG vs. CG | 2 | 234 | 0.28 | -1.88, 2.43 | 0.80 | <0.00001 | 98% |
| EG, before vs. after | 2 | 234 | -1.57 | -2.81, -0.33 | 0.01 | <0.0001 | 94% |
| CG, before vs. after | 2 | 234 | -1.06 | -1.30, -0.83 | <0.00001 | 0.39 | 0% |
| CG (control group), the group with ETV monotherapy; EG (experimental group), the group with ETV plus Tα1 combination therapy; SMD, standardized mean difference; CI, confidence interval; ALT, alanine aminotransferase; ALB, albumin; TBIL, total bilirubin; AST, aspartate aminotransferase; A/G, the albumin globulin ratio. | | | | | | | |
