# Supplementary material for: The clinical efficacy and adverse effects of Entecavir plus Thymosin alpha-1 combination therapy versus Entecavir Monotherapy in HBV-related cirrhosis: a systematic review and meta-analysis
Source: BMC Gastroenterol. 2020 Oct 19;20:348. doi: 10.1186/s12876-020-01477-8 (PMC7574490; doi:10.1186/s12876-020-01477-8)
Supplement: Supplementary file 7 — Additional file 7: Table S3. Summary of pooled results regarding meta-regression. [file 12876_2020_1477_MOESM7_ESM.doc]

**Table S3. Summary of pooled results regarding meta-regression**

| Variable | P | | |
| --- | --- | --- | --- |
|  | Year of publication | Type of cirrhosis | Criteria of diagnostic |
| ALT | | | |
| After treatment, EG vs. CG | 0.328 | 0.757 | 0.899 |
| EG, before vs. after | 0.161 | 0.247 | 0.015 |
| CG, before vs. after | 0.482 | 0.584 | 0.050 |
| TBIL | | | |
| After treatment, EG vs. CG | 0.552 | 0.520 | 0.918 |
| EG, before vs. after | 0.413 | 0.657 | 0.082 |
| CG, before vs. after | 0.700 | 0.496 | 0.063 |

CG (control group), the group with ETV monotherapy; EG (experimental group), the group with ETV plus Tα1 combination therapy; ALT, alanine aminotransferase; TBIL, total bilirubin.
